# Motor Functional Evaluation from Physiology and Biomechanics to Clinical and Training Application

**DOI:** 10.1155/2015/542496

**Published:** 2015-12-01

**Authors:** Jullia Maria D'Andréa Greve, Angelica Castilho Alonso, Luiz Mochizuki, Paulo R. Lucareli, Chandramouli Krishnan, Richard Baker

**Affiliations:** ^1^University of São Paulo, São Paulo, Avenida Pacaembu 1777, 01234-001 São Paulo, SP, Brazil; ^2^The University Nove de Julho, São Paulo, Brazil; ^3^University of Michigan, Ann Arbor, USA; ^4^University of Salford, Lancashire, UK

This special issue was proposed to combine biology, biomechanics, and human locomotion and the use of this knowledge to improve rehabilitation; prescription of physical activity for health promotion and for sports performance are important for the science, not only for the gait but for every type of movement and exercise.

In this special issue, men and women were compared in sports, physical activity, and standing posture. P. G. Mouroço and colleagues show that men and women achieve fast front crawl swimming with different strategies; while men use arm stroke, women swim fast using whole-body mean forces. M. Persiani and colleagues showed that men consistently had larger COP parameters than women. They applied lateral optic flow perturbances and found that they cause asymmetry in postural balance and different lateralization of postural controls in men and women. H. Jaafar and colleagues evaluated force-velocity relationship during cycling and arm cranking exercise. They compared the reliability of maximal power, maximal pedal rate, and maximal force in active men and women and showed that they are different according to sex. It is interesting to see how close or far human performance is according to biology.

In rehabilitation, evaluation of functional movements is the key element in building a rehabilitation program. M. Caimmi and colleagues provide a set of normative data from healthy subjects of different ages doing upper-limb functional tasks (reaching and hand-to-mouth movements). Their results are important for upper-limb evaluation in different populations. Normative data in quiet standing is important as well. Therefore, A. C. Alonso and colleagues showed that anthropometric variables can explain part of center of pressure sway quiet standing.

Developing techniques to evaluate body motion during walking is necessary to understand how people move. C. Buckley and colleagues measured upper body movement during walking and associated it with postural control in persons with Parkinson's disease (PD). They showed that persons with PD attenuate body accelerations differently compared to controls. E. Bergamini and colleagues proposed a method to evaluate wheelchair propulsion based on biomechanics and successfully applied the results to regulate a training program for young wheelchair athletes.

But technology is not only applied in rehabilitation to improve measurements; it is applied as an intervention. C. Luque-Moreno and colleagues reviewed and compared VR intervention in stroke, looking for frequently used outcome measures. Multimodal approach, combining VR and conventional physiotherapy, presented the best results in balance and gait. VR mostly improves gait speed, balance, and motor function in stroke patients.

Exercise-based interventions improve performance. N. Hedayatpour and D. Falla reviewed neuromuscular adaptations to eccentric exercise, depicting histochemical, metabolic, and neural adaptations due to eccentric training. Besides, exercise changes human biology. W. Y. V. Mak and W. K. C. Lai showed that short duration of resistance exercise may provoke a transient increase in central arterial stiffness in healthy young men. They analyzed acute effect of Valsalva manoeuvre during resistance exercise on arterial stiffness.

So, this special issue is very rich and joins different views of the motion study in the same publication, empowering the knowledge for all professionals of this area.

*Jullia Maria D'Andréa Greve* *Angelica Castilho Alonso* *Luiz Mochizuki* *Paulo R. Lucareli* *Chandramouli Krishnan* *Richard Baker*

